# Supplementary material for: 2,6-Diaminopurine as a highly potent corrector of UGA nonsense mutations
Source: Nat Commun. 2020 Mar 20;11:1509. doi: 10.1038/s41467-020-15140-z (PMC7083880; doi:10.1038/s41467-020-15140-z)
Supplement: Supplementary file 1 — Supplementary Information [file 41467_2020_15140_MOESM1_ESM.pdf]

Supplementary information for:

2,6-Diaminopurine as a highly potent corrector of UGA nonsense mutations

Trzaska et al.

Supplementary Methods

DAP identification

Identity between natural and commercial synthetic DAP samples was ascertained from:
- a comparison of the HPLC chromatograms obtained for i) the H7 extract, ii) commercial synthetic DAP, and iii) the H7 extract co-injected with commercial DAP;
- a comparison of the MS/MS spectra, showing identical fragmentation patterns (characteristic m/z ions) obtained for commercial DAP, the DAP peak selected in the H7 extract, and the isolated natural DAP;
- a comparison of the ^{1}H and ^{13}C NMR data obtained in the ^{1}H-1D spectra and the ^{1}H-^{13}C HMBC 2D spectra, showing the long-range correlations between ^{1}H and ^{13}C signals characteristic of the DAP structure.

HPLC method

HPLC chromatograms were obtained with a liquid chromatography system (321 pump, Gilson) coupled to a diode array detector (170 DAD, Gilson). They were processed with Unipoint software (Gilson).
Conditions used: injection volume 60 µL, flow rate 0.8 ml/min, detection at 254 nm; oven (CIO-10AS, Shimadzu) temperature : 308.15 K; column Acclaim™ Polar advantage II, Thermo Scientific, C18, 5 µm, 120 Å, 4.6x250 mm; elution solvents: A: milliQ water + 0.1% formic acid; B: acetonitrile + 0.1% formic acid.
Elution gradient:

Time (min)	solvent A (%)	solvent B (%)
0	100	0
8.5	100	0
22.5	60	40
24.5	60	40
26	100	0

NMR spectrometry

The spectra for DAP identification were obtained on a 600 MHz Avance III HD spectrometer equipped with a TCI cryoplatform (Bruker Biospin).

Mass spectrometry

MS-MS fragmentation was done on a quadripole-time of flight (Q-TOF) hybrid mass spectrometer equipped with an electrospray ionization source (Compact, Bruker). The data were analyzed with Compass Hystar 4.1 software. CID spectrum conditions: mass range 50-1300 m/z; positive electrospray ionization mode; collision energy 30 eV.

Transcriptomic analysis

Ten million HeLa cells were exposed to DMSO or DAP for 24 h. Total RNA was extracted with RNazol reagent. Libraries were prepared by poly(A) enrichment with a PolyA Spin mRNA Isolation Kit (Biolabs) and strand-specific cDNA libraries were prepared with the Illumina

Truseq Stranded mRNA Sample Preparation Kit (Illumina Inc., USA). Reads were sequenced on an Illumina HiSeq 2500 device which produced six libraries of paired-end 100-nt long reads for three biological replicates of the two conditions. Reads were mapped with BWA-MEM (version 0.7.12-r1039, http://bio-bwa.sourceforge.net/) against the human genome (GRCh37.p13). Reads having passed the default Illumina filter procedure (chastity filter) were counted on human genes with featureCounts (version 1.5.1, http://subread.sourceforge.net/). Condition clustering was checked by principal component analysis with R package FactoMineR (version 1.36). Differential expression analysis was performed on the raw read counts with R package DESeq2 (version 1.14.1) [1]. DEGs were determined with an adjusted p-value (Student's t-test) ≤ 0.05 and a |log2FC| >=1. Heatmap representations of global gene expression of each library were produced using the normalized read counts (varianceStabilizingTransformation function of DESeq2) with R function heatmap.2 from R package gplots (version 3.0.1). Enrichment in Gene Ontology terms was determined on the set of DEGs on the Gorilla website (cbl-gorilla.cs.technion.ac.il – last update of human database on Jun 8, 2019) [2, 3].

Two-dimensional differential gel electrophoresis

2D-DIGE was performed on a mixture of protein samples labeled according to the CyDye minimal labeling method (GE Healthcare). Control, H7, and DA extracts were labeled respectively with Cy2, Cy3, and Cy5 and pooled. Finally, 5.4 µL Destreak Reagent (GE Healthcare), 1% (v/v) IPG buffer pH 3 − 11NL (GE Healthcare) and CHAPS lysis buffer were added to reach a final volume of 450 µl. Migration in the first dimension, i.e. isoelectric focusing (IEF), was carried out with pH 3 − 11NL, 18 cm IPG strips (GE Healthcare) after overnight passive rehydration. IEF was carried out on an Ettan IPGphor 3 IEF unit (GE Healthcare) with the following parameters: (1) constant voltage of 150 V for 3 h, (2) constant voltage of 300 V for 3 h, (3) gradient from 300 V to 1000 V over 6 h, (4) gradient from 1000 V to 10000 V over 1 h, and (5) constant voltage of 10000 V for 2 h. The temperature was set at 20°C and the current was limited to 50 µA/strip. After the first dimension, the strips were equilibrated for 15 min in equilibration buffer containing 50 mM Tris-HCl pH 8.8, 6 M urea, 30% (v/v) glycerol, 2% (w/v) SDS, 0.02% Bromophenol Blue supplemented with 1% (w/v) DTT and subsequently for 15 min in equilibration buffer supplemented with 2.5% (w/v) iodoacetamid. The strips were rinsed in cathode buffer, placed on top of the second-dimension gel, and sealed with low-melt agarose. Cathode and anode buffers were added in the electrophoresis tank (Ettan DALT six, GE Healthcare) and the gels were run at 20°C. The migration settings were: (1) 5 mA for 2 h and (2) 25 mA until the sample reached the end of the gel. Gels were scanned with a Typhoon FLA 9500 (GE Healthcare).

Toxicity assay measuring propidium iodide incorporation This toxicity assay was performed 24 h after cell treatment. HeLa cells present in the cell culture medium and HeLa cells attached to the dish were collected. After 5 min of centrifugation at 300xg, the supernatant was removed and the cell pellet resuspended in annexin binding buffer component C, before addition of propidium iodide according to the manufacturer's protocol (Life Technologies). After a 5-minute incubation at room temperature, dead cells were counted with a TALI cytometer (Life Technologies).

MTT colorimetric toxicity assay After 24 h of DMSO, DAP, or STS treatment, HeLa cells were incubated in cell culture medium containing 0.5 mg/ml 3-(4,5-dimethylthiazol-2-yl)-2,5-diphenyltetrazolium bromide (MTT) for 2 h at 37°C. The cells were then incubated for 30 min at 37°C in the solubilization solution (isopropanol/1N HCl 23:1) before collecting the supernatant to measure the absorbance at 540 and 620 nm for the background.

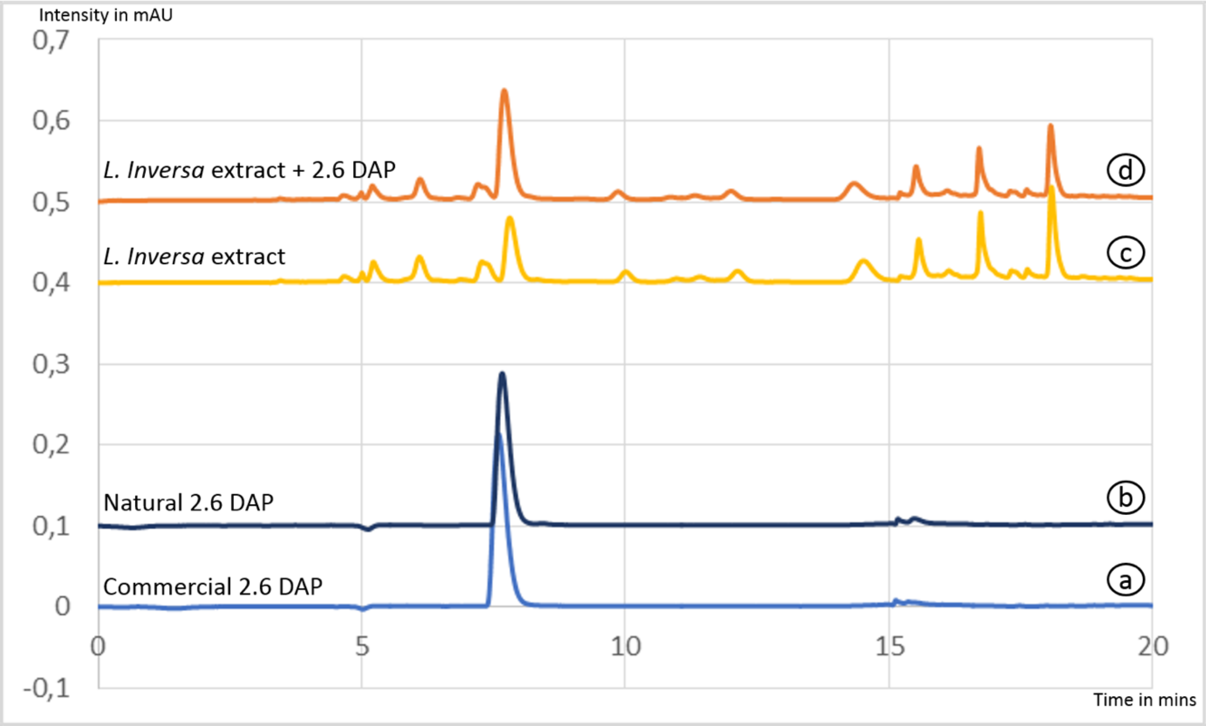

Supplementary Figure 1. Identification of the 2,6-diaminopurine in H7 extract. RP-HPLC elution profiles of commercial (a) and natural (b) 2,6-DAP samples and of *L. inversa* extract H7 (c) and *L. inversa* extract H7 co-injected with commercial DAP (d), showing the presence of DAP (Rt: 7.8 min) in extract H7 (C18, 5 µm analytical column Acclaim Polar Advantage II (4.6 x 250 mm) eluted for 26 min with a linear gradient from 0.1% formic acid in H_2O to 0.1% formic acid in acetonitrile; detection at 254 nm).

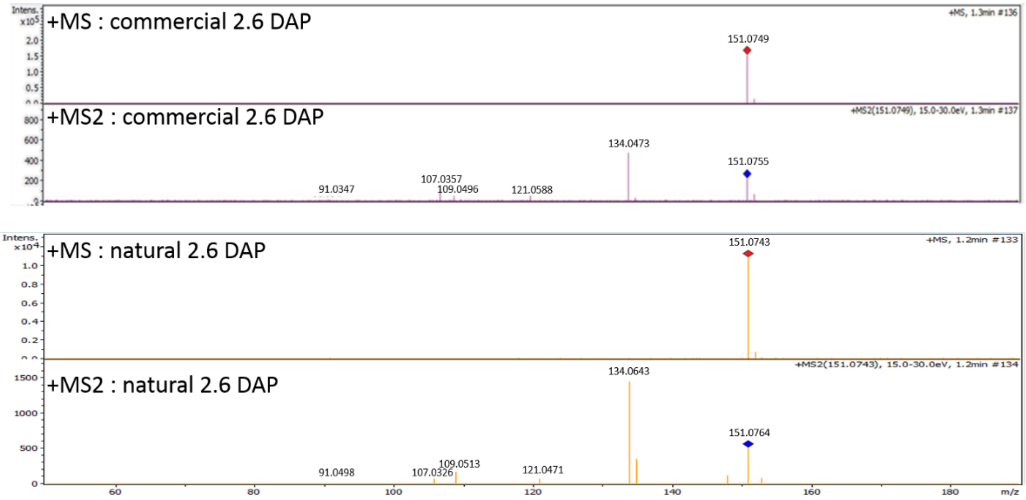

Supplementary Figure 2. Characterization of 2,6-DAP by mass spectrometry. Mass spectra (MS) showing the MH^{+} ions and CID fragmentation patterns (MS2) of commercial and natural DAP samples.

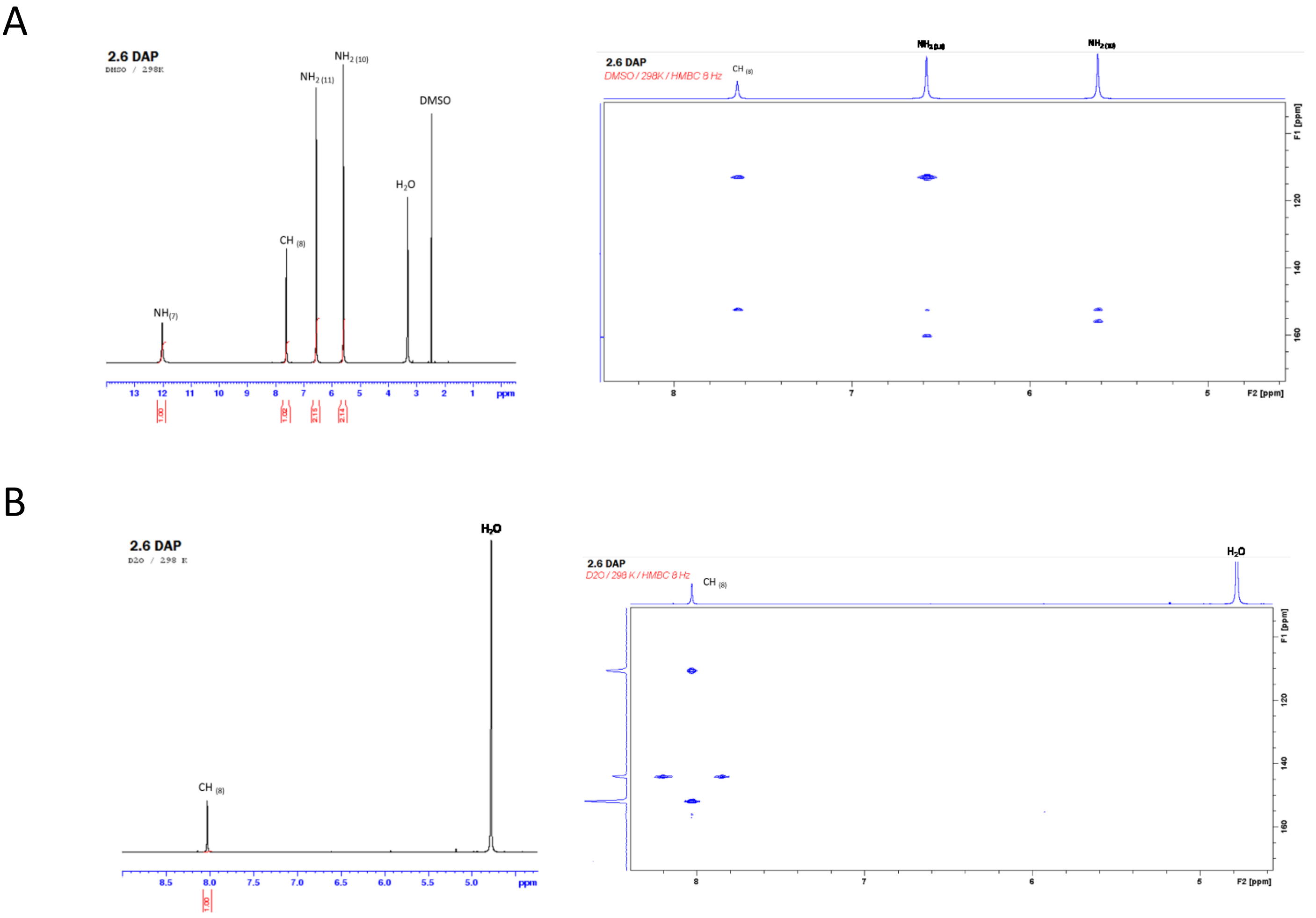

Supplementary Figure 3. Characterization of 2,6-DAP by ^{1}H and ^{13}C NMR (600 MHz, 298K). ^{1}H 1D (left panels) and ^{1}H-^{13}C HMBC spectra (8 Hz) (right panels) obtained in DMSO-d6 (A) and D_2O (B).

A

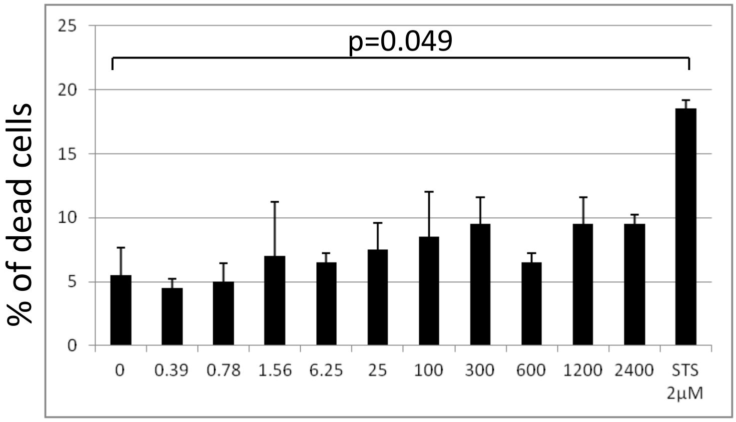

B

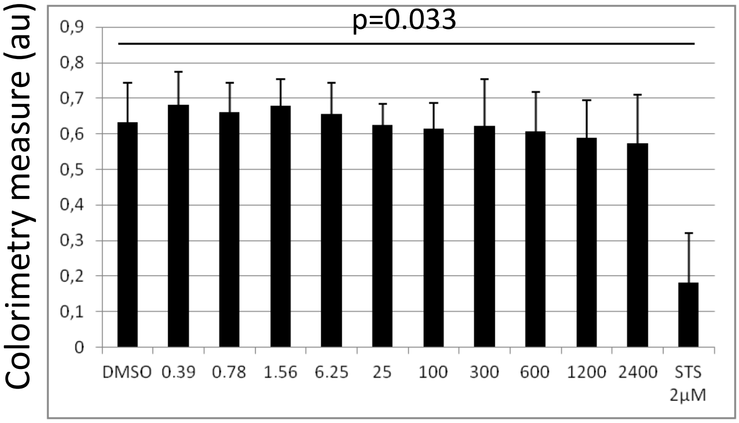

Supplementary Figure 4. DAP is not toxic. (A) HeLa cells were incubated with propidium iodide after treatment for 24 h with DMSO (0), 0.39 µM to 2400 µM DAP, or 2 µM apoptosis inducer staurosporine (STS). (B) HeLa cells were incubated with MTT after treatment for 24 h with DMSO (0), 0.39 µM to 2400 µM DAP, or 2 µM staurosporine (STS). The results presented are representative of two independent experiments at least. Error bar= S.D., p-values (p) were calculated using Student's t-test. Source data are provided as a Source Data file.

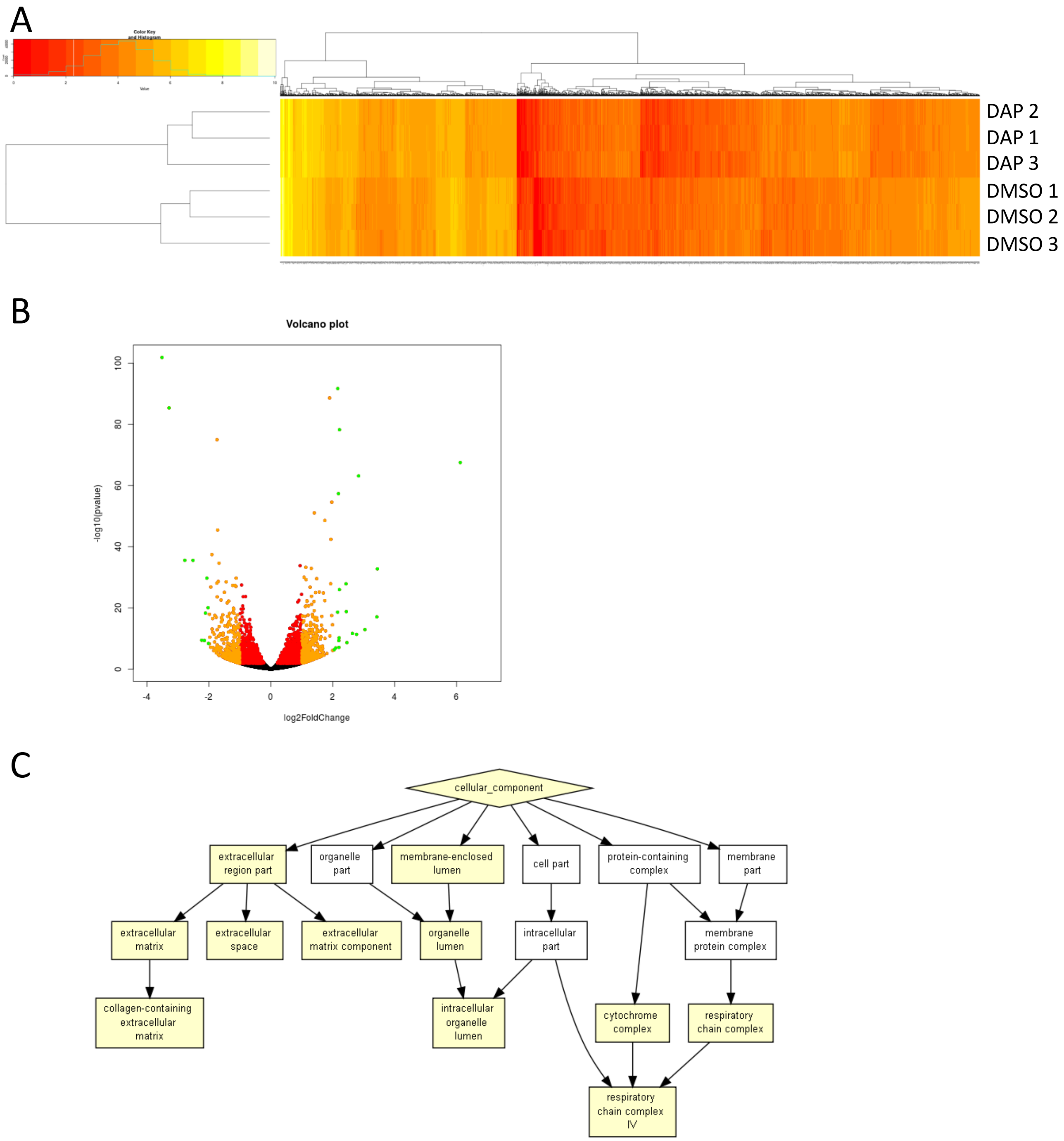

Supplementary Figure 5. DAP has a middle impact on the global cell transcription profile. (A) Heatmap representation of up- and down-regulated genes, showing a weak effect of DAP on HeLa cells in three independent experiments. The 3228 shown genes (adjusted p-value (Student's t-test) < 0.05) are represented by normalized read counts with a log2 scale (see Supplementary Material). Dendrograms show the separation of DMSO-treated (DMSO1-3) vs. DAP-treated (DAP 1-3) libraries without any clear pattern of dysregulation between samples. (B) Volcano plot of statistical significance against fold-change between Control VS DAP treatments (red: adjusted p-value (Student's t-test) <0,05; orange: adjusted p-value (Student's t-test) <0,05 and absolute log2FC >1; green: adjusted p-value (Student's t-test) <0,05 and absolute log2FC >2). (C) Schematic representation of the GO analysis of the pathways dysregulated in the presence of DAP. Source data are provided as a Source Data file.

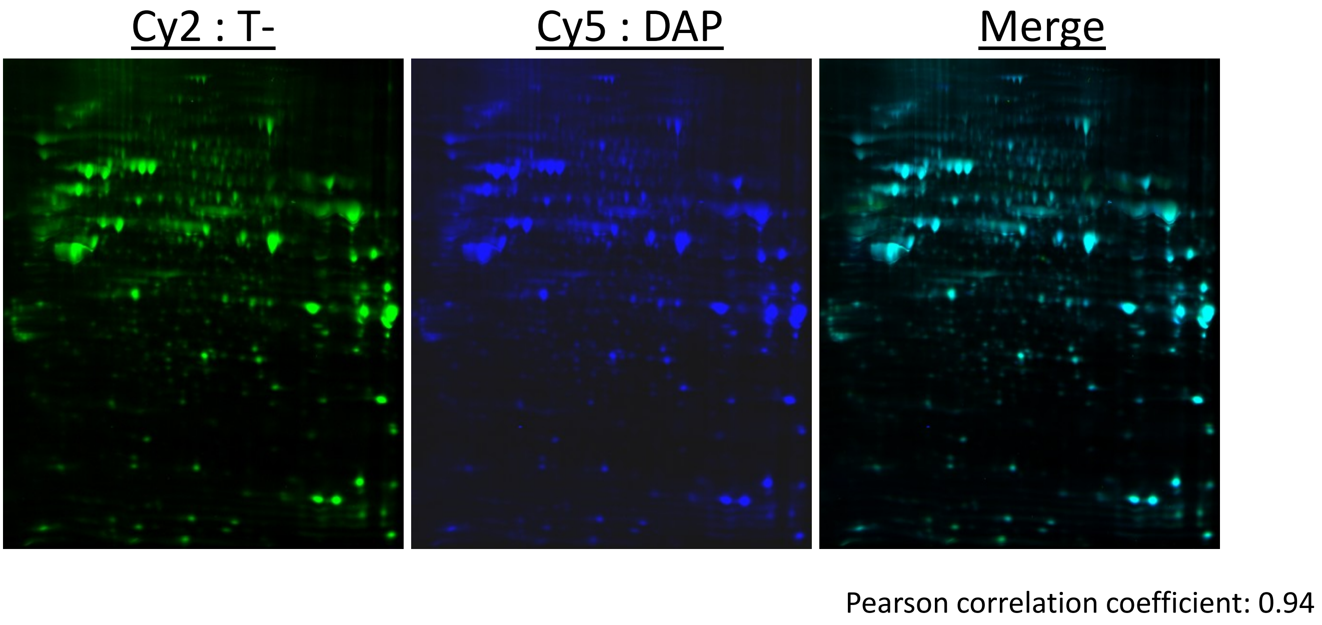

Supplementary Figure 6. 2D-DIGE analysis. Extracts of HeLa cells exposed to DMSO (T) or DAP (DAP) were loaded for 2D-DIGE. Cy2 was used to detect proteins from cells exposed to DMSO (left gel) and Cy5 was used to detect proteins from cells exposed to DAP (middle gel). An overlay of Cy2 and Cy5 gels is presented on the right side (Merge). Pearson correlation coefficient was calculated using Imaris analysis.

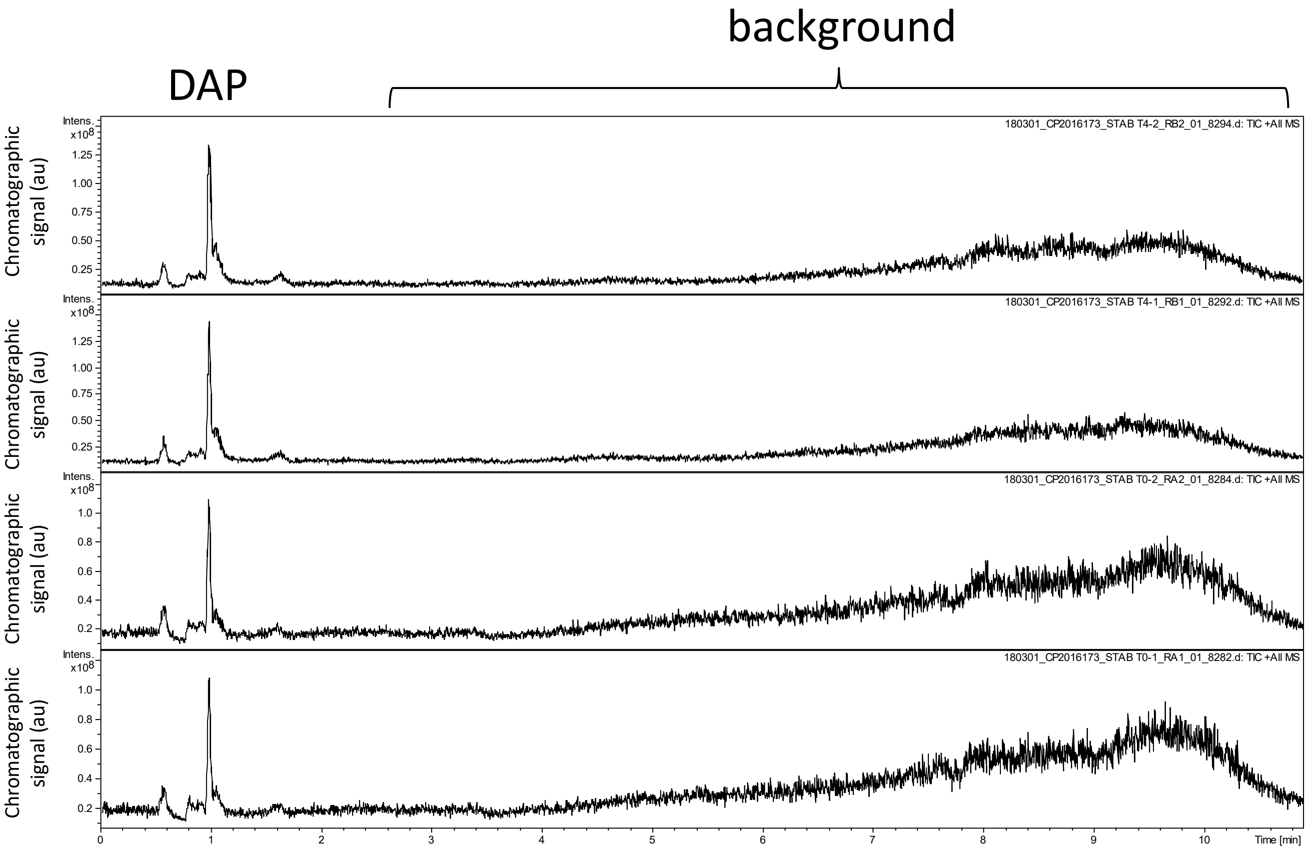

Supplementary Figure 7. DAP is stable in hepatic extract. Chromatograms illustrating the stability of DAP after 4 h of incubation in S9 mix (in duplicate, two upper panels) as compared to the amount of DAP extracted at T0 (in duplicate, two lower panels).

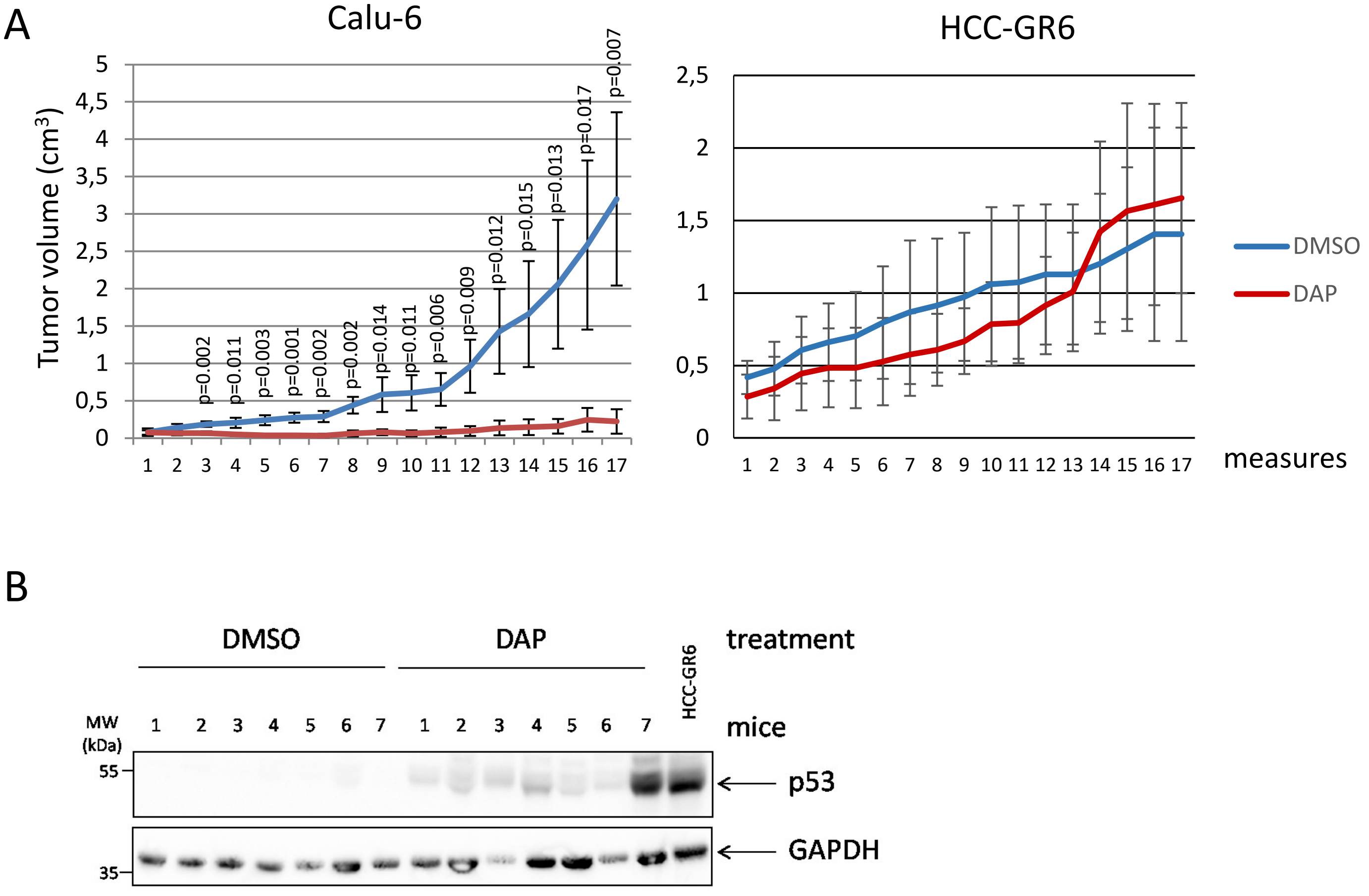

Supplementary Figure 8. DAP corrects UGA nonsense mutations *in vivo*. (**A**) 28 nude mice were injected with Calu-6 (left panel) or HCC-GR6 (right panel) cells to promote tumor development. The mice were exposed to DMSO or DAP for about five weeks and tumor size was measured three times weekly and plotted on the graph. For each experimental condition, 7 mice were used. (B) Western blot analysis of p53 protein in the Calu-6-cell tumors of the seven DMSO-exposed and seven DAP-exposed mice. GAPDH was used as loading control. A molecular weight marker is indicated to the left the gel. Error bar= S.D., p-values (p) were calculated using Student's t-test. Source data are provided as a Source Data file.

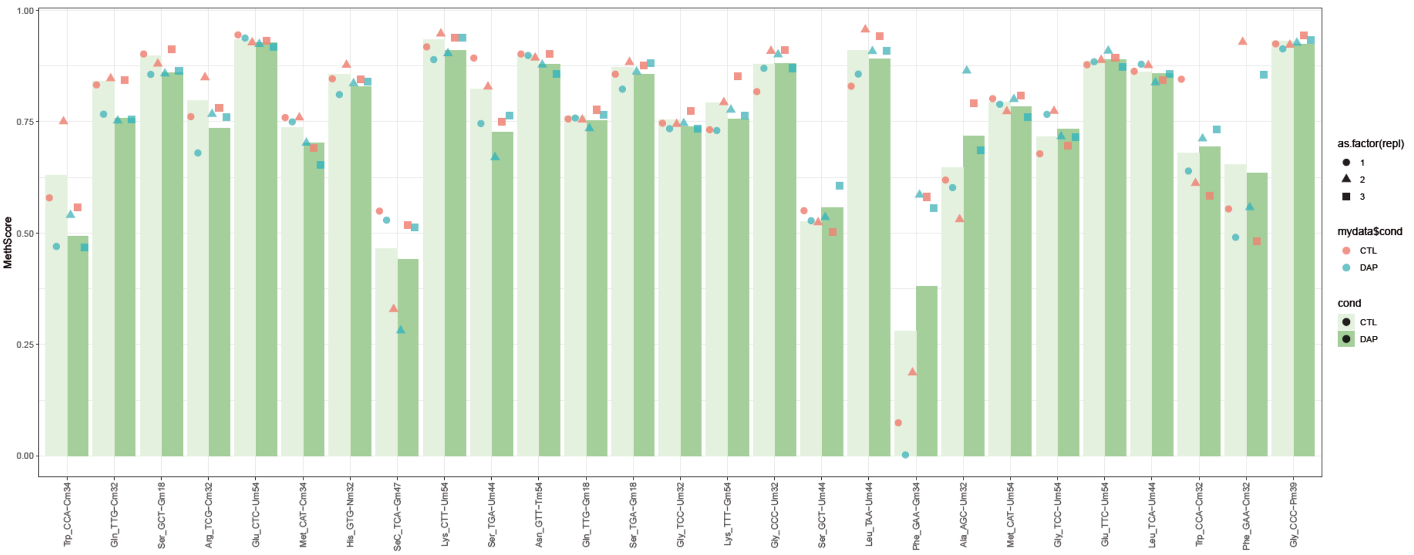

Supplementary Figure 9. Influence of DAP on tRNA modifications: 2’-O-methylation analysis of tRNAs by RiboMethSeq. A MethScore was attributed to each tRNA 2’-O-methylation in tRNA purified from HeLa cells treated with DAP (dark green histogram) or DMSO (light green histogram). Source data are provided as a Source Data file.

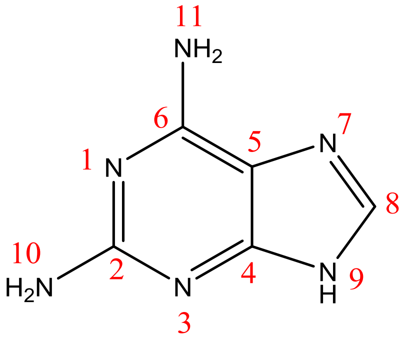

	In DMSO d_6		In d_2O	
N°	δ_H	δ_C	δ_H	δ_C
2		160.0		
4		152.1		151.8
5		112.8		110.5
6		155.7		155.9
8	7.66 (s)	135.2	8.03 (s)	143.9
NH (9)	12.06 (s)			
NH_2(10)	5.63 (s)			
NH_2(11)	6.60 (s)			

Supplementary Table 1. ^{1}H and ^{13}C chemical shift values of DAP obtained in DMSO-d6 and D_2O (600 MHz, 298K).

	UAA	UAG	UGA
DMSO	373±59	364.5±9	458.25±19
G418 1mg/m	495±34	2275.5±805	8466.25±478
H7 10ng/µl	1282.75±259	508.25±22	33049.25±1771
F7 10ng/µl	384.25±55	416.75±9	440.75±49
F8 10ng/µl	370.75±31	394.75±40	442.75±40
F9 10ng/µl	370.5±68	386±3	796.5±324
F10 10ng/µl	456±89	392.75±5	1865.25±1120
F11 10ng/µL	338±28	414.25±55	447±16
F12 10ng/µl	353.5±86	379.5±37	552±89
F13 10ng/µl	462.5±98	449.5±18	5567.25±148
F14 10ng/µl	327±68	410±105	495.25±22
F15 10ng/µl	403.5±28	386±40	2908±1039
DAP 3.75µM	370.75±86	366.5±43	20599.75±1015
H7 100ng/µl	3920±3712	807±321	76940.5±44413
F7 100ng/µl	355.75±83	405.75±55	443±83
F8 100ng/µl	368.75±58	390.5±34	386.25±71
F9 100ng/µl	351±40	384±61	1874±873
F10 100ng/µl	355.5±40	388±43	25119.75±4908
F11 100ng/µl	329.75±65	386±16	464.75±28
F12 100ng/µl	357.5±37	388.25±37	488.75±37
F13 100ng/µl	1106.25±225	493±80	26916±2085
F14 100ng/µl	362±49	405.75±37	565±52
F15 100ng/µl	999±419	492.75±49	25660.75±7203
DAP 37.5µM	610.75±408	451.5±145	35939.75±1176

Supplementary Table 2: Quantification of the luciferase activity measured in the presence of H7 extract, fractions or molecules (G418, DAP) corresponding to the readthrough activity on UAA, UAG or UGA premature stop codons.

	DAP	G418
DMSO	793±289	601±14
0.39µM	962±318	690±16
0.78µM	1241±372	772±15
1.56µM	1730±503	841±17
6.25µM	6071±1660	1004±19
25µM	40668±10487	1000±19
100µM	299032±73740	1120±20
300µM	224429±47425	1702±31
600µM	115880±26184	1217±21

Supplementary Table 3: Quantification of the luciferase activity measured in the presence of increasing amounts of DAP or G418 corresponding to the efficiency of UGA premature stop codon readthrough .

List of the 20 genes showing at least 4-fold upregulation in the presence of DAP

Gene Name	GeneID	log2FoldChange	p-value	Gene function
S100A5	6276	6.119	3.080E-68	S100 family member involved in Ca2+ intracellular regulation
CMYA5	202333	3.442	1.899E-33	Anchor protein of PKA
DCHS1	8642	3.427	9.008E-18	Cadherin family member
LRIT3	345193	3.040	1.221E-13	Immunoglobulin-like protein
STC2	8614	2.836	7.358E-64	Secreted protein putatively involved in Ca and phosphate transport
GRK7	131890	2.771	4.546E-12	Retina-specific kinase
IL18BP	10068	2.635	2.408E-12	inhibitor of the proinflammatory cytokine IL18
ITGA10	8515	2.458	2.317E-09	integrin involved in cell adhesion
ACRC	93953	2.441	1.663E-19	putative nuclear protein
TRIB3	57761	2.430	1.419E-28	putative protein kinase induced by the transcription factor NF-kappaB
ASNS	440	2.217	5.830E-79	Asparagine synthetase
ZNF721	170960	2.216	1.117E-26	putative transcriptional regulator
PNLDC1	154197	2.206	6.267E-11	PARN Like, Ribonuclease Domain Containing 1
NUP210L	91181	2.200	8.162E-08	nucleoporin 210 like
ZNF460	10794	2.196	5.326E-10	Zinc finger protein
ANKRD11	29123	2.186	4.476E-58	protein interacting with histone deacetylases
SPEN	23013	2.164	2.006E-92	hormone inducible transcriptional repressor
SESN2	83667	2.158	2.522E-19	member of the sestrin family of PA26-related proteins
CNTF	1270	2.096	1.291E-07	neurotrophic factor
ADAM32	203102	2.049	4.829E-07	member of the disintegrin family

List of the 10 genes showing at least 4-fold downregulation in the presence of DAP

Gene Name	GeneID	log2FoldChange	p-value	Gene function
COX2	4513	-3.516	1.361E-102	protein involved in the synthesis of prostaglandins
COX3	4514	-3.286	4.176E-86	COX-3 is an enzyme encoded by the PTGS1 gene
ND6	4541	-2.779	2.717E-36	This protein is not functional in humans
COX1	4512	-2.516	2.860E-36	subunit of the respiratory chain Complex I
SYNC	81493	-2.233	3.857E-10	Cytochrome c oxidase subunit I
HSD17B8	7923	-2.144	4.807E-10	member of the intermediate filament family
ATP8	4509	-2.113	5.218E-19	Estradiol 17 beta-dehydrogenase 8
OLR1	4973	-2.065	1.925E-30	mitochondrial ATP synthase Fo subunit 8
INSL4	3641	-2.029	9.034E-21	Oxidized low-density lipoprotein receptor 1
IDH2	3418	-2.015	4.480E-09	insulin-like 4 protein Isocitrate dehydrogenase

Supplementary Table 4: Results of DESeq2 for the DEGs

Supplementary references

1. Love MI, Huber W, Anders S. Moderated estimation of fold change and dispersion for RNA-seq data with DESeq2. *Genome Biol* **15**, 550 (2014).

2. Eden E, Navon R, Steinfeld I, Lipson D, Yakhini Z. GOrilla: a tool for discovery and visualization of enriched GO terms in ranked gene lists. *BMC Bioinformatics* **10**, 48 (2009).

3. Eden E, Lipson D, Yogev S, Yakhini Z. Discovering motifs in ranked lists of DNA sequences. *PLoS Comput Biol* **3**, e39 (2007).

4. Benjamini Y, Hochberg Y. Controlling the False Discovery Rate: A Practical and Powerful Approach to Multiple Testing. *Journal of the Royal Statistical Society Series B (Methodological)* **57**, 289-300 (1995).
