# Supplementary material for: 2,6-Diaminopurine as a highly potent corrector of UGA nonsense mutations
Source: Nat Commun. 2020 Mar 20;11:1509. doi: 10.1038/s41467-020-15140-z (PMC7083880; doi:10.1038/s41467-020-15140-z)
Supplement: Supplementary file 3 — Reporting Summary [file 41467_2020_15140_MOESM3_ESM.pdf]

nature research

Corresponding author(s): Lejeune Fabrice

Last updated by author(s): Jan 24, 2020

Reporting Summary

Nature Research wishes to improve the reproducibility of the work that we publish. This form provides structure for consistency and transparency in reporting. For further information on Nature Research policies, see Authors & Referees and the Editorial Policy Checklist.

Statistics

For all statistical analyses, confirm that the following items are present in the figure legend, table legend, main text, or Methods section.

n/a	Confirmed	
☐	☒	The exact sample size (*n*) for each experimental group/condition, given as a discrete number and unit of measurement
☐	☒	A statement on whether measurements were taken from distinct samples or whether the same sample was measured repeatedly
☐	☒	The statistical test(s) used AND whether they are one- or two-sided<br>*Only common tests should be described solely by name; describe more complex techniques in the Methods section.*
☒	☐	A description of all covariates tested
☒	☐	A description of any assumptions or corrections, such as tests of normality and adjustment for multiple comparisons
☐	☒	A full description of the statistical parameters including central tendency (e.g. means) or other basic estimates (e.g. regression coefficient) AND variation (e.g. standard deviation) or associated estimates of uncertainty (e.g. confidence intervals)
☐	☒	For null hypothesis testing, the test statistic (e.g. *F*, *t*, *r*) with confidence intervals, effect sizes, degrees of freedom and *P* value noted<br>*Give P values as exact values whenever suitable.*
☒	☐	For Bayesian analysis, information on the choice of priors and Markov chain Monte Carlo settings
☒	☐	For hierarchical and complex designs, identification of the appropriate level for tests and full reporting of outcomes
☒	☐	Estimates of effect sizes (e.g. Cohen's *d*, Pearson's *r*), indicating how they were calculated

Our web collection on statistics for biologists contains articles on many of the points above.

Software and code

Policy information about availability of computer code

Data collection	No software was used.
Data analysis	For the transcriptomic analysis reads were sequenced on an Illumina HiSeq 2500 device which produced six libraries of paired-end 100-nt long reads for three biological replicates of the two conditions. Reads were mapped with BWA-MEM (version 0.7.12-r1039, http://bio-bwa.sourceforge.net/) against the human genome (GRCh37.p13). Reads having passed the default Illumina filter procedure (chastity filter) were counted on human genes with featureCounts (version 1.5.1, http://subread.sourceforge.net/). Condition clustering was checked by principal component analysis with R package FactoMineR (version 1.36). Differential expression analysis was performed on the raw read counts with R package DESeq2 (version 1.14.1). Differentially expressed genes were determined with an adjusted p-value >0.05 and a |log2FC| >2. Heatmap representation of global gene expression of each library have been produced using the normalized read counts (varianceStabilizingTransformation function of DESeq2) with R function heatmap.2 from R package gplots (version 3.0.1).

For manuscripts utilizing custom algorithms or software that are central to the research but not yet described in published literature, software must be made available to editors/reviewers. We strongly encourage code deposition in a community repository (e.g. GitHub). See the Nature Research guidelines for submitting code & software for further information.

Data

Policy information about availability of data

All manuscripts must include a data availability statement. This statement should provide the following information, where applicable:

- Accession codes, unique identifiers, or web links for publicly available datasets
- A list of figures that have associated raw data
- A description of any restrictions on data availability

All data described in the manuscript are fully available

Field-specific reporting

Please select the one below that is the best fit for your research. If you are not sure, read the appropriate sections before making your selection.

☒ Life sciences ☐ Behavioural & social sciences ☐ Ecological, evolutionary & environmental sciences

For a reference copy of the document with all sections, see nature.com/documents/nr-reporting-summary-flat.pdf

Life sciences study design

All studies must disclose on these points even when the disclosure is negative.

Sample size	All figures were based on at least two independent experiments excepted for figures 2E and S6 that were performed only once because figure 2E uses patient cells and we ethically could not request to collect from the same patient two times cells but we provide results from three different patients. For Figure S6, we estimated that the analysis was on all protein spots making each spot independent from others. Finally, for the figure 4, we used 7 mice for each conditions because it is the smallest number of mice to provide statistically significant results and respect the ethical request of using animal number as low as possible.
Data exclusions	No data were excluded.
Replication	All experiments were reproduced with similar results.
Randomization	We used cell lines carrying endogenous nonsense mutations.
Blinding	Blinding was not relevant to our study because we had to chose genes carrying UGA nonsense mutations to characterize DAP.

Reporting for specific materials, systems and methods

We require information from authors about some types of materials, experimental systems and methods used in many studies. Here, indicate whether each material, system or method listed is relevant to your study. If you are not sure if a list item applies to your research, read the appropriate section before selecting a response.

Materials & experimental systems

n/a	Involved in the study	
☐	☒	Antibodies
☐	☒	Eukaryotic cell lines
☒	☐	Palaeontology
☐	☒	Animals and other organisms
☒	☐	Human research participants
☒	☐	Clinical data

Methods

n/a	Involved in the study	
☒	☐	ChIP-seq
☒	☐	Flow cytometry
☒	☐	MRI-based neuroimaging

Antibodies

Antibodies used	anti p53 (Santa Cruz Biotechnologies-sc126-DO1-H0610 or Biorad VMA00019); anti CBP80 (Santa Cruz Biotechnologies-sc48803-H300-F2909);anti GAPDH (Santa Cruz Biotechnologies-sc32233-6C5-K3016);anti Tubulin (Abcam-ab134185--0606151330;anti FTSJ1 (Sigma-AU49131--QC20453); anti CTU1 (Abcam-ab136083--GR3210741 1); anti UPF1 (Abcam-ab86057--GR64008 4); anti UPF3X (Abcam-ab134566--GR248830); anti Y14 (Santa Cruz Biotechnologies-sc32312-4C4-E1705); anti Importin9 (Abcam-ab52605--YI0808025)
Validation	anti UPF2 (generous gift from Pr. Lynne Maquat referenced in Lejeune et al.,2003-Molecular Cell, Vol. 12, 675–687)

Eukaryotic cell lines

Policy information about cell lines

Cell line source(s)	All cell lines were purchased from ATCC.
Authentication	None of the cell lines used were authentified.
Mycoplasma contamination	Mycoplasma are not present in our cell culture because all cell lines are cultured in the presence of Zellshield a regeant preventing mycoplasma contamination.
Commonly misidentified lines (See ICLAC register)	None of the used cell lines belong to the ICLAC register.

Animals and other organisms

Policy information about studies involving animals; ARRIVE guidelines recommended for reporting animal research

Laboratory animals	6-weeks old Nude mice from Charles River laboratory were used in this study.
Wild animals	The study did not involved any wild animal.
Field-collected samples	Lepista flaccida mushroom was collected in various forest around Paris and in particular in Compiègne forest.
Ethics oversight	Animal experimentation protocol was evaluated by the ethical comittee CEEA Nord-Pas-de-Calais and provided the authorization CEEA112009R.

Note that full information on the approval of the study protocol must also be provided in the manuscript.
