# Supplementary material for: 2,6-Diaminopurine as a highly potent corrector of UGA nonsense mutations
Source: Nat Commun. 2020 Mar 20;11:1509. doi: 10.1038/s41467-020-15140-z (PMC7083880; doi:10.1038/s41467-020-15140-z)
Supplement: Supplementary file 4 — Description of Additional Supplementary Files [file 41467_2020_15140_MOESM4_ESM.pdf]

Description of Additional Supplementary Files

File Name: Supplementary Data 1

Description: Results of GOrilla for the DEGs. 698 of the 775 DEGs are associated with a GO term. BP Biological Process; MF Molecular Function; CC Cellular Component; N total number of genes; B total number of associated genes in the GO term; n number of genes at the top of the user's input list or in the target set when appropriate; b number of genes in the intersection. Enrichment is the result of the calculation (b/n) / (B/N). FDR q-value is the correction of the p-value (Wald test) for multiple testing using the Benjamini and Hochberg method 4.

4. Benjamini Y, Hochberg Y. Controlling the False Discovery Rate: A Practical and Powerful Approach to Multiple Testing. *Journal of the Royal Statistical Society Series B (Methodological)* **57**, 289-300 (1995).
